# Advertisement of unhealthy commodities in Bristol and South Gloucestershire and rationale for a new advertisement policy

**DOI:** 10.1186/s12889-023-15995-z

**Published:** 2023-06-05

**Authors:** Lauren J. Scott, James Nobles, Carlos Sillero-Rejon, Rowan Brockman, Zoi Toumpakari, Russell Jago, Steven Cummins, Sarah Blake, Jeremy Horwood, Frank de Vocht

**Affiliations:** 1 National Institute for Health Research Applied Research Collaboration West (NIHR ARC West), Bristol, UK; 2 Population Health Sciences, Bristol Medical School, University of Bristol, Canynge Hall, 39 Whatley Road, Bristol, BS8 2PS UK; 3 University Hospitals Bristol and Weston NHS Foundation Trust, Bristol, UK; 4 Obesity Institute, School of Health, Leeds Beckett University, Leeds, UK; 5 Centre for Exercise, Nutrition & Health Sciences, School for Policy Studies, University of Bristol, Bristol, UK; 6 Department of Public Health, Environments & Society, Faculty of Public Health and Policy, London School of Hygiene and Tropical Medicine, London, UK

**Keywords:** Outdoor advertising, Unhealthy commodities, Health inequalities, Policy implementation, Mixed methods, HFSS

## Abstract

**Background:**

Bristol City Council introduced a new advertisement policy in 2021/2022 which included prohibiting the advertising of unhealthy food and drink (HFSS), alcohol, gambling and payday loans across council-owned advertising spaces. This mixed methods study is part of the BEAR study, and aimed to explore the rationale and the barriers and facilitators to implementing the policy, and describe the perceived advertising environment prior to implementation.

**Methods:**

Semi-structured interviews were carried out with seven stakeholders involved in the design and implementation of the advertising policy. A stakeholder topic guide was developed before interviews took place to help standardise the lines of inquiry between interviewees. A resident survey was developed to collect socio-demographic data and, for the purpose of this study, information regarding observations of advertising for HFSS products, alcohol and gambling.

**Results:**

Fifty-eight percent of respondents residing in Bristol and South Gloucestershire reported seeing advertisements for unhealthy commodities in the week prior to completing the survey. This was highest for HFSS products (40%). 16% of residents reported seeing HFSS product advertisements specifically appealing to children. For HFSS products in particular, younger people were more likely to report seeing adverts than older people, as were those who were from more deprived areas. An advertisement policy that restricts the advertisement of such unhealthy commodities, and in particular for HFSS products, has the potential to reduce health inequalities. This rationale directly influenced the development of the advertisement policy in Bristol. Implementation of the policy benefitted from an existing supportive environment following the ‘health in all policies’ initiative and a focus on reducing health inequalities across the city.

**Conclusions:**

Unhealthy product advertisements, particularly for unhealthy food and drinks, were observed more by younger people and those living in more deprived areas. Policies that specifically restrict such advertisements, therefore, have the potential to reduce health inequalities, as was the hope when this policy was developed. Future evaluation of the policy will provide evidence of any public health impact.

**Supplementary Information:**

The online version contains supplementary material available at 10.1186/s12889-023-15995-z.

## Introduction

The Commercial Determinants of Health are defined by the World Health Organization as “private sector activities that affect people’s health positively or negatively” [1], while others emphasise their negative impact as “strategies and approaches used by industry to promote products and choices that are detrimental to health” [2]. These unhealthy commodity industries (UCIs) typically include the manufacture, marketing and selling of tobacco, alcohol, and foods and drinks high in fat, salt or sugar (HFSS), but more recently have expanded to include gambling [3] and payday loans. Advertising is an important strategy utilised by UCIs to influence awareness, attitudes and preferences, purchase intent, purchase requests, purchase, and consumption of unhealthy commodities [4].

Outdoor spaces (e.g. bus stops and billboards) and transport facilities are important advertising locations. In 2021, the UK outdoor advertising market generated approximately £900 million in revenue [5]. Outdoor advertising is thought to reach 98% of the UK population at least once a week [6]. For example, data from Scotland indicates that HFSS products totalled about 33% of all “out-of-home” bus stop advertisements, alcohol 4%, and gambling 0.4%, [7]. Data from Northern England similarly indicate that about half of all advertisements on bus stops were for food and beverages, of which 35% were considered less healthy [8]. Self-reported data from London indicate as much as 85% of survey respondents report exposure to HFSS product advertising in the past seven days [9]. Evidence suggests that unhealthy commodity advertising has cumulative effects, especially on children and adolescents, in that attitudes as well as consumption behaviours correlate with the frequency of exposure to marketing messages [10–12]. Further, it has been shown that people from disadvantaged households have greater exposure to outdoor and recreational settings, exacerbating existing health inequalities [13].

Population exposure to unhealthy commodity advertisements is recognised as a modifiable risk factor for the development of non-communicable diseases and has been identified as a priority area for policy action particularly for alcohol and tobacco, and foods and non-alcoholic beverages marketing to children and adolescents, by the World Health Organization [14]. It is, therefore, a logical hypothesis that reducing exposure through the reduction or removal of advertisements might reduce unhealthy behaviours and subsequently reduce such disease. Evidence from modelling studies suggests that unhealthy commodity advertising restrictions can have beneficial reductions in the purchase and consumption of such products; for example, a 15% reduction in the quantity of crisps sold [15] and a 5–8% reduction in alcohol consumption [16] might be possible. Further, modelling of the potential impacts of the Transport for London (TfL) advertisement restriction policy in London [9], which restricted HFSS product advertising across their entire network from February 2019, indicated it might have resulted in 4.8% fewer individuals with obsesity, and reduce the incidence of diabetes and cardiovascular disease by 2,857 and 1,915 individuals within 3 years post intervention, and could save the NHS £218 million [17].

The evaluation of the TfL advertising restriction showed that over a 10-month follow-up period there was a 6.7% (1,001 kcal [456–1,546]) reduction in purchasing of calories from HFSS products per household. The largest effect was observed for chocolate and confectionery (-19%; 318 kcal [200–436]), which is of particular interest because this advertising mostly disappeared following the restriction as there was a lack of policy-compliant substitutes (in contrast to, for example, sugary drinks for which zero or low-calorie alternatives are available). Importantly, the observed changes were larger in more deprived households, households with children, and households where the main food shopper was overweight or obese, indicating the policy may be well targeted to high-risk groups and may have the potential to reduce health inequalities. Indeed, the accompanying health economic evaluation concluded based on extensive modelling that greater benefits, a 37% higher gain in quality-adjusted life-years, were expected in the most socioeconimically deprived groups compared to the least deprived [17].

Bristol City Council has a long-term “One City Plan” which engages public and private sector organisations, large charities, voluntary groups, and grassroots communities to deliver a fairer, healthier and safer city. A key part of this plan was an Advertising and Sponsorship Policy which identifies restrictions for advertising and/or sponsorship in council-owned advertising spaces and which, with respect to place-based outdoor advertising encompassed 861 advertising spaces at 283 bus stops. This was introduced in November 2021 with an expectation of becoming fully embedded by summer 2022, once existing contracts ended. This policy is more restrictive than the TfL restriction and prohibits the promotion of HFSS products and their brands (unless they advertise non-HFSS alternatives), alcoholic drinks, tobacco or tobacco substitute products, weapons, gambling, illegal drugs, and high-cost short-term loans.

The current study aims to describe, through a mixed methods design, (i) the history and development of the Bristol Advertising and Sponsorship Policy and the main facilitators and barriers to implementation, and (ii) inequalities in exposure to the advertisement of unhealthy commodities in Bristol and a comparator area, South Gloucestershire. This study is part of the BEAR (Bristol Evaluation of Advertisement Restrictions) study, which aims to evaluate the impact of this advertisement policy on public health.

## Methods

### Study design and setting

Between January and March 2022, we conducted semi-structured interviews with a range of stakeholders and collected baseline data through a cross-sectional survey of a representative sample of Bristol (intervention area) and the adjacent area of South Gloucestershire (comparator area) residents. These data were collected after the policy had been introduced, but prior to most of the contracts ending (most ended in April 2022), so largely still represented the lay of the land before the policy had been implemented. Based on the 2021 census, Bristol is a city of 472,400 residents in the South-West of England, and South Gloucestershire is a neighbouring area with 290,400 residents [18]. South Gloucestershire has large rural parts but also includes several towns and urban areas, having merged with the northern and eastern areas of Bristol.

### Qualitative data collection and analysis

Semi-structured interviews were carried out with seven stakeholders directly involved in the design and implementation of the advertising policy. Stakeholders worked in the council (*n* = 4), voluntary sector organisations (*n* = 2), or were a local councillor (*n* = 1). Interviewees were purposefully sampled based on their involvement in the policy and through relationships held across the wider project team. Several project team members were involved in the planning of the policy and so were able to create links between the researchers and interviewees. Initial contact was made via email with prospective participants, providing an information sheet and a consent form.

A stakeholder topic guide (Online Supplementary Materials (OSM) Table S1) was developed before interviews took place to help standardise the lines of inquiry between interviewees. The interviews aimed to gather stakeholder views on: how they had been involved in the policy; the history of the policy; the process of planning the policy; the anticipated impacts of the policy; initial challenges and facilitators facing the design and implementation of the policy; and, future plans for the policy. As with the survey, the interviews took place after the policy had been introduced, but prior to full implementation. All interviews were carried out online, using Zoom or Microsoft Teams, by an experienced qualitative researcher (JN or CSR), audio recorded and transcribed verbatim. Interviews lasted between 30 and 90 min.

Framework analysis was used [19] to code and organise the data on an interviewee-by-interviewee basis so that comparisons could be drawn between responses. Framework analysis was appropriate here as it allowed us to interpret data both within and between interviewees, and to do so in a time efficient and robust manner [19]. One researcher (RB) led the analysis, with JN and CSR contributing to this process through discussion at regular intervals. Transcripts were initially read by RB to help with familiarisation of the data. The research team then created a deductive analytical framework based on the focus of inquiry, which was was imported into Microsoft Excel in the form of a matrix. Interviews were coded against a set of high-order themes within the matrix on an interviewee-by-interviewee basis. In short, this helped to organise the interview data and retain key participant quotes alongside a summary of the researcher interpretation. In the last step, the researcher refined the final themes and sub-themes to delineate patterns between interviewees. The final themes and subthemes, alongside the reasoning for the development of these themes, were agreed upon by the three researchers. To ensure that our analysis was a valid representation of the processes followed to design and implement the policy, we shared our initial themes and reports with interviewees for comment. Two interviewees suggested several minor changes to describe more accurately some of the bureaucratic processes that the policy went through. Illustrative quotes are used throughout to support themes and sub-themes.

### Quantitative survey data collection and analysis

We co-developed a survey with the Councils to collect socio-demographic data including age, sex, ethnicity, disability, household set-up, occupation, bus use, postcode, and whether respondents had been in their local area during the week before completing the survey. The main survey included questions regarding observations of advertising for HFSS products, alcohol and gambling, as well as locations of such advertising (bus stops, billboards, etc.) and self-reported consumption of such products (consumption data presented elsewhere) [20]. All questions concerned advertising in their local area the week before questionnaire completion. We purposely did not define local area more specifically than ‘your street and surrounding streets’ as we recognise any more comprehensive definition might confuse, and possibly still not cover all possibilities. For reference, the survey is provided in OSM Table S2.

In Bristol, the online survey was sent to all participants of the Bristol Citizens’ Panel, a volunteer panel of citizens that broadly represents the demographics of Bristol and who regularly complete surveys about matters concerning local policies and issues, using the Panel’s standard communication method of a link to the survey provided in an email [21]. The sample was supplemented by subscribers to the Panel *Ask Bristol* newsletter (~ 3,000 people) and stakeholder contacts (~ 200 equality organisations and partner organisations). Additionally, paper copies were sent to the most deprived 20% of communities and provided at libraries and on request to digitally-excluded citizens and others who requested it. Alternative formats were provided to people with specific accessibility needs. Together these formed the basis of the survey methodology routinely used by the Council to get information from a representative sample of the Bristol population. In the control area of South Gloucestershire, the survey was distributed using similar methods, which included sending to all participants in the comparable South Gloucestershire Viewpoint Panel [22], which currently has approximately 2,300 participants and also a paper distribution to 10,000 residential addresses proportionately represented by ward, electronically. This was supplemented by promotion of the survey to equalities organisations and through the council newsletter ‘South Glos eNews’ (which has approximately 75,000 subscribers) and social media.

Data were collated by the councils in the two areas; postcodes were translated to Lower Super Output Areas (LSOAs) and all identifying information was removed before data transfer to the University of Bristol for analysis. Index of multiple deprivation (IMD) was mapped to participants using their LSOAs. All data are binary or categorical (except age) and are presented as counts and/or percentages. Age was categorised as follows: 18–34 years, 35–44 years 45–64 years, 65 + years. South Gloucestershire used previously collected demographic data based on fewer ethnic groups. To match data from the different sources, therefore, categories for ethnicity were condensed into two categories: White and non-white. Many questions had multiple options and a final option for ‘none of the above’. Due to conflicting information (i.e. participants selecting an option and also selecting ‘none of the above’), the ‘none of the above’ options were not used and instead, a ‘yes to any’ variable was generated for each of these questions: ‘yes’ if any of the multiple options were ticked, and ‘no’ if none of the options were ticked. Respondents were split into Bristol and South Gloucestershire residents based on their LSOAs, irrespective of which survey they completed. Respondents who resided in LSOAs outside of Bristol and South Gloucestershire (or with missing LSOA data) were excluded prior to any analysis (*n* = 143). Similarly, respondents who reported they had been out of area all week in the week prior to questionnaire completion (or did not complete this question) were excluded (*n* = 110). Finally, respondents who were younger than 18 years old were excluded (*n* = 17). Chi-squared tests were used to calculate *p*-values for the differences between advertising in different demographic groups. ONS Census 2021 data was explored to assess how comparable the sample was to the populations of Bristol and South Gloucestershire.

## Results

### Qualitative findings

We developed four main themes and a series of associated sub-themes to support the study aims. These themes include: 1) history of the policy; 2) the process of planning the policy; 3) the anticipated impacts of the policy; and 4) the barriers and facilitators to the policy implementation. Sub-themes are underlined within the text.

### History of the policy

Interviewees stated that an *extensive* advertising policy was not in place prior to the Bristol Advertising and Sponsorship Policy. That said, there was a commitment by the council to embed health into all policies, with a core focus on reducing health inequalities across the city. Importantly, this was supported by the city Mayor. The council had also recently signed up to a national healthy weight initiative which required the support of local leaders (from the council and the NHS). As such, interviewees stated that there was already a *supportive climate in Bristol* for such a policy.

*“...there was a general head of steam building about the problems with advertising” – Participant 7, Bristol City Council*

Outside of the council, a *third sector organisation had campaigned* in Bristol about the harms associated with unhealthy commodity advertising. Their intention was to make senior leaders, and the public, more aware of these harms. This campaigning included writing to all councillors within Bristol. Further, there was the *TfL policy precedent* which contributed to the ambition of Bristol City Council to design and implement their own policy (See Barriers and Facilitators theme).

*“I think it was as a result of there being a precedent nationally so the fact that London had brought in the policy, it meant that it’s easier for others” – Participant 4, Voluntary Sector Organisation*

### Planning the policy

The policy took approximately 18 months to develop. Interviewees indicated that the first 12 months were used to draft the policy, albeit the actual time required to write the policy was minimal and was seen as a “back burner” task. The last 6 months represented a concerted effort to engage wider stakeholders and move the policy through the decision-making processes. Collectively, and with the input of Public Health colleagues, an evidence- and business-case for the policy was created. Interviewees stated that it was easier to build the case for the policy because of the “groundwork” done by the TfL team.

*“I would say actually in terms of policy development it was probably relatively light touch actually!” – Participant 2, Bristol City Council*

The policy then passed through the council approvals process. Interviewees described how an internal communications plan was created to help council staff to operationalise the policy. Within the plan, it also clarified who was accountable for the day-to-day administration of the policy; much of which rested with the internal communication team and the private-sector organisation that manages the advertising spaces on the council’s behalf.

### Anticipated impacts

A *reduction in the purchase of HFSS products* was the main anticipated impact. Interviewees believed that this would occur more in inner-city areas, which house more deprived neighbourhoods, and consequently where advertising prevalence was greatest. Some interviewees, therefore, went further to suggest that this would help *reduce health inequalities*. Longer-term, several interviewees suggested that this would contribute to *lower levels of obesity*, *improved population health*, and *reduced demand for health and social care*.

*“I would expect... a reduction in HFSS advertising, particularly in busy areas...children from more deprived neighbourhoods are more exposed to HFSS advertising because they tend to live near those busier areas...and so...I would expect to see a reduction in health inequalities much later on down the line but earlier on I would also expect to see a reduction in purchasing based on the TfL data” – Participant 4, Voluntary Sector Organisation*

However, other interviewees questioned how likely these impacts would be. Firstly, some thought that companies would *adapt their advertising* and either increase the non-HFSS products in their portfolio (i.e. healthier substitutes) or move more of their advertising online. Secondly, others thought the *policy would have a limited impact* due to the volume of advertising space held by the council. It was estimated that about 30% of all outdoor advertising space in Bristol was council owned. There were inconsistent views on the impact that the policy may have on *revenue generated through advertising*.

*“People use the word ban, but this is a substitution policy so McDonalds can rock up and advertise a black coffee and a Greek salad...they can’t do the golden arches by themselves, and you can’t do the massive burgers” – Participant 2, Bristol City Council*

*“My instinct would be that…they will find some other way to advertise to people. So… you will see an increase or spike in them advertising online… to…the audiences they wanted to reach” – Participant 3, Bristol City Council.*

*“It’s not necessarily the case that a junk food advertising ban will create lots of immediate financial benefits but it’s a step in the right direction of a bigger health landscape” – Participant 6, Voluntary Sector Organisation*

### Initial barriers and facilitators to the policy implementation

Given that the interviews were carried out between January and March 2022, the policy had started to be implemented as new advertising contracts were rolled out. Therefore, we were able to ask interviewees about the initial barriers and facilitators to the policy implementation.

Multiple interviewees highlighted that the culture within Bristol, across the council and among the wider public is supportive of progressive policies. This likely made it easier for the policy to pass through the council bureaucracy. Additionally, the council stated that there was a collective vision for how they wanted to address health inequalities and that the advertising policy would contribute towards this rather than push against it.

*“Bristol’s a very radical place. The population are consistently ahead of the politicians in terms of the policies they want to see” – Participant 7, Bristol City Council*

The early work in developing the policy was made easier because of the TfL precedent, also an important point noted earlier regarding the history of the policy. This included guidance on how to operationalise more complicated aspects of the policy (e.g. the Nutrient Profile Model). Similarly, some interviewees thought that the policy was straightforward for companies to adapt their advertising to; the idea that “no brand is banned” meant that products, rather than companies, were directly impacted.

There were several barriers mentioned that challenged the initial design, implementation, and efficacy of the advertising policy. The first was that because the council owned a relatively small proportion of the advertising space in Bristol (the majority of spaces were privately owned), the efficacy of the restriction, in terms of its reach and impact was questioned. Interviewees stated that the council had very little to no control over what was advertised in privately owned space. Then, from a implementation perspective, there was no dedicated project officer to help implement and monitor the adherence to the policy.

*“We have things like the Coca Cola bus that will suddenly appear ...in the city and we have absolutely no jurisdiction if they don’t park on council property” – Participant 1, Bristol City Council*

Some interviewees highlighted the conflict between the objectives of this policy to restrict alcohol advertisement and other council departments' objectives such as those related to stimulating the city's economy through leisure, particularly nighttime and festivals.

*“[A local leader] wanted us to do a map of night time economy businesses, so is it okay for us to include pubs on that map. I don’t know.” – Participant 3, Voluntary Sector Organisation*

*“the last time I went to the [local festival] they had these huge, sweet stalls which take up half the road...the problem is it brings in money to the city.” – Participant 1, Bristol City Council*

### Quantitative survey findings

We received 2,813 completed questionnaires. After removing 39 who resided outside of our two areas, 104 with missing address information, 77 who were out of area all week, 33 who were missing this information, and 17 who were younger than 18 years old, we included 2,543 responses for analysis (1,110 from Bristol and 1,433 from South Gloucestershire).

Across both councils, respondents were more often female (59%), of white ethnicity (89%), and over 45 years (79%). Respondents in South Gloucestershire were older (53% vs. 31% 65 + years), more likely to live as a couple (47% vs. 31%), more likely to be retired (53% vs. 32%) and less deprived (79% vs. 22% IMD decile 6–10), than Bristol respondents (Table 1). Seventy-four percent of respondents in Bristol and 61% in South Gloucestershire reported using the bus, which is relevant as bus stops make up the majority of the council owned advertisement spaces.

**Table 1:** Respondent characteristics

	**Bristol (*n* = 1,110)**		**South Gloucestershire (*n* = 1,433)**		**Overall (*n* = 2,543**^**a**^**)**	
	**n**	**%**	**n**	**%**	**n**	**%**
**Sex**						
Female	620	55.9%	876	61.1%	1496	58.8%
Male	457	41.2%	542	37.8%	999	39.3%
Other	5	0.5%	1	0.1%	6	0.2%
Missing	28	2.5%	14	1.0%	42	1.7%
**Age**						
18–34 years	170	15.3%	78	5.4%	248	9.8%
35–44 years	159	14.3%	104	7.3%	263	10.3%
45–64 years	427	38.5%	476	33.2%	903	35.5%
65 + years	342	30.8%	760	53.0%	1102	43.3%
Missing	12	1.1%	15	1.0%	27	1.1%
**Ethnicity**						
White	982	88.5%	1292	90.2%	2274	89.4%
Non-white	95	8.6%	86	6.0%	181	7.1%
Missing	33	3.0%	55	3.8%	88	3.5%
**Household**						
Lives alone	370	33.3%	370	25.8%	740	29.1%
Couple	343	30.9%	667	46.5%	1010	39.7%
Family	335	30.2%	372	26.0%	707	27.8%
Sharers	52	4.7%	10	0.7%	62	2.4%
Other	5	0.5%	3	0.2%	8	0.3%
Missing	5	0.5%	11	0.8%	16	0.6%
**Employment**						
Full time	349	31.4%	349	24.4%	698	27.4%
Part time	166	15.0%	172	12.0%	338	13.3%
Self-employed	74	6.7%	79	5.5%	153	6.0%
Unemployed	77	6.9%	20	1.4%	97	3.8%
Retired	359	32.3%	758	52.9%	1117	43.9%
Student	23	2.1%	7	0.5%	30	1.2%
Other/combination	60	5.4%	32	2.2%	92	3.6%
Missing	2	0.2%	16	1.1%	18	0.7%
**IMD decile**						
1 (most deprived)	598	53.9%	0	0.0%	598	23.5%
2	58	5.2%	8	0.6%	66	2.6%
3	60	5.4%	60	4.2%	120	4.7%
4	78	7.0%	125	8.7%	203	8.0%
5	72	6.5%	106	7.4%	178	7.0%
6	35	3.2%	120	8.4%	155	6.1%
7	74	6.7%	208	14.5%	282	11.1%
8	51	4.6%	179	12.5%	230	9.0%
9	47	4.2%	167	11.7%	214	8.4%
10 (least deprived)	37	3.3%	460	32.1%	497	19.5%
Missing	0	0.0%	0	0.0%	0	0.0%
**Bus use**						
Daily	76	6.8%	2	0.1%	78	3.1%
Several times per week	143	12.9%	61	4.3%	204	8.0%
Several time per month	213	19.2%	156	10.9%	369	14.5%
Once a month or less	384	34.6%	631	44.0%	1015	39.9%
Never	285	25.7%	558	38.9%	843	33.1%
Missing	9	0.8%	25	1.7%	34	1.3%

*IMD* Index of Multiple Deprivation

^a^because respondents younger than 18 years of age (*n* = 17) were removed from these analyses, the sample size differs slightly from those in [20]

Based on data from the 2021 census, the adult population of Bristol was 51% female, 41% under 35 years, 16% 65 + years, and 83% White ethnicity. The adult population of South Gloucestershire was 51% female, 30% under 35 years, 23% 65 + years, and 92% White ethnicity [18]. Comparable data was not available on IMD. Our survey sample is therefore representative in terms of ethnicity, but has a higher percentage of women and older people compared to the populations in these areas.

**Fig. 1:**
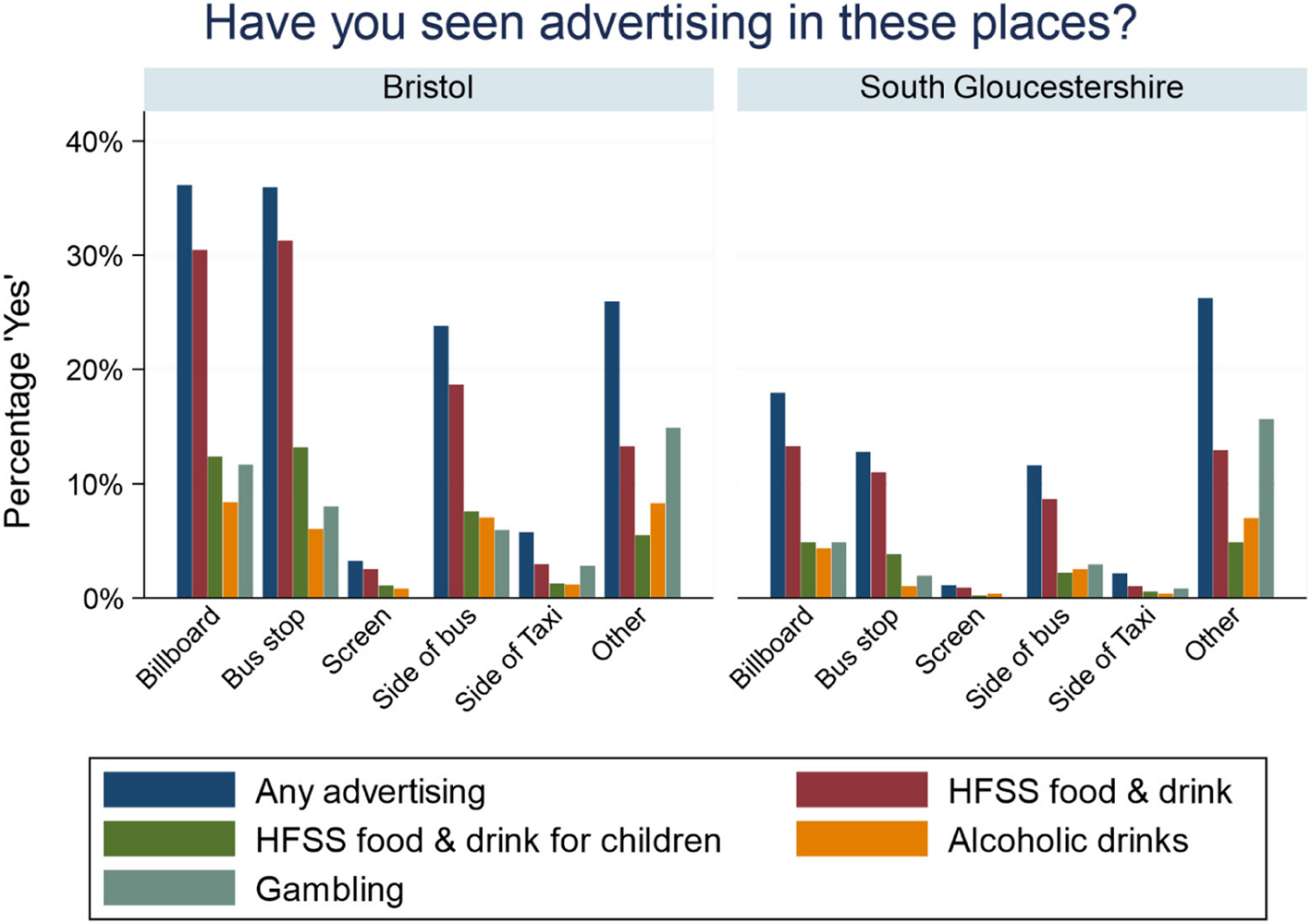
Reported placement of advertisements HFSS = Food and drinks high in fat, salt and/or sugar

In the week prior to completing the questionnaire, 58% of respondents reported observing some kind of unhealthy advertising; 40% of respondents reported observing advertising for any HFSS, 17% for alcoholic drinks, 21% for establishments selling alcohol and 28% for gambling (Table 2). The most observed advertising for HFSS products was for fast food (34%), followed by sugary drinks (15%) and chocolate & sweets (14%); 60% of respondents reported seeing no HFSS advertising at all (Table 2). This pattern was similar for HFSS adverts directed at children, although numbers were lower across all products (Table 2). The fast-food chain with the highest observed advertising was McDonald’s (33%), followed by KFC (18%), coffee chains (16%), Dominos (16%) and Subway (15%). The most often reported advertisements for alcoholic drinks were those for beer/lager/cider (11%), followed by spirits (9%) and wine (8%); 83% of respondents did not report observing any alcohol advertising (Table 2). The most observed gambling adverts were for the National Lottery (15%), Ladbrokes (9%), online gambling (8%) and William Hill (7%); 72% did not observe any gambling advertisements (Table 2). For almost all measured advert types, South Gloucestershire respondents reported seeing less advertising than Bristol respondents (Table 2). Billboards and bus stops were the most common places to see advertising, followed by the sides of buses; advertising was reportedly observed very little on screens or sides of taxis. Patterns, but not percentages per se, were comparable for Bristol and South Gloucestershire (Fig. 1).

**Table 2:** Self-reported advert exposure

	**Bristol (*n* = 1,110)**		**South Gloucestershire (*n* = 1,433)**		**Overall (*n* = 2,543)**	
	**n**	**%**	**n**	**%**	**n**	**%**
**Overall**						
Any unhealthy commodities	733	66.0%	748	52.2%	1481	58.2%
**HFSS food & drink**						
Any HFSS food & drink	584	52.6%	444	31.0%	1028	40.4%
Chocolate/Sweets	188	16.9%	167	11.7%	355	14.0%
Biscuits/cake	130	11.7%	111	7.7%	241	9.5%
Desserts	117	10.5%	86	6.0%	203	8.0%
Sugary cereal	89	8.0%	66	4.6%	155	6.1%
Crisps/savoury snacks	160	14.4%	114	8.0%	274	10.8%
Fast food	526	47.4%	348	24.3%	874	34.4%
Sugary drinks	229	20.6%	154	10.7%	383	15.1%
**HFSS food & drink for children**						
Any HFSS food & drink for children	254	22.9%	156	10.9%	410	16.1%
Chocolate/Sweets	101	9.1%	72	5.0%	173	6.8%
Biscuits/cake	63	5.7%	31	2.2%	94	3.7%
Desserts	55	5.0%	29	2.0%	84	3.3%
Sugary cereal	61	5.5%	33	2.3%	94	3.7%
Crisps/savoury snacks	69	6.2%	40	2.8%	109	4.3%
Fast food	204	18.4%	107	7.5%	311	12.2%
Sugary drinks	134	12.1%	63	4.4%	197	7.7%
**Fast-food chains**						
Any Fast-food chains	657	59.2%	597	41.7%	1254	49.3%
McDonald’s	490	44.1%	350	24.4%	840	33.0%
Burger King	172	15.5%	75	5.2%	247	9.7%
KFC	286	25.8%	160	11.2%	446	17.5%
Subway	195	17.6%	179	12.5%	374	14.7%
Dominos	159	14.3%	238	16.6%	397	15.6%
Papa Johns	52	4.7%	70	4.9%	122	4.8%
Nando’s	18	1.6%	32	2.2%	50	2.0%
Greggs	155	14.0%	147	10.3%	302	11.9%
Pret a Manger	13	1.2%	7	0.5%	20	0.8%
Coffee chains	160	14.4%	245	17.1%	405	15.9%
Other	52	4.7%	46	3.2%	98	3.9%
**Alcoholic drinks**						
Any alcoholic drinks	234	21.1%	186	13.0%	420	16.5%
Beer/lager/cider	153	13.8%	121	8.4%	274	10.8%
Wine	104	9.4%	91	6.4%	195	7.7%
Spirits	141	12.7%	99	6.9%	240	9.4%
Mixtures (e.g. alcopops)	45	4.1%	25	1.7%	70	2.8%
Other	15	1.4%	10	0.7%	25	1.0%
**Establishments serving alcohol**						
Any establishments serving alcohol	196	17.7%	340	23.7%	536	21.1%
Pubs	162	14.6%	301	21.0%	463	18.2%
Restaurants	66	5.9%	103	7.2%	169	6.6%
Other	28	2.5%	37	2.6%	65	2.6%
**Gambling companies**						
Any gambling companies	372	33.5%	352	24.6%	724	28.5%
Sport betting	61	5.5%	48	3.3%	109	4.3%
Ladbrokes	129	11.6%	93	6.5%	222	8.7%
Betfair	77	6.9%	68	4.7%	145	5.7%
Paddy Power	79	7.1%	58	4.0%	137	5.4%
William Hill	110	9.9%	72	5.0%	182	7.2%
National Lottery	186	16.8%	201	14.0%	387	15.2%
Casinos	39	3.5%	11	0.8%	50	2.0%
Bingo venues	73	6.6%	37	2.6%	110	4.3%
Online gambling	112	10.1%	78	5.4%	190	7.5%
Racecourses	20	1.8%	9	0.6%	29	1.1%
Other	61	5.5%	66	4.6%	127	5.0%

*HFSS* Food and drink high in fat, salt and/or sugar

Younger respondents were more likely to observe advertising than older respondents (77% in 18–34 year olds vs. 53% in 65 + year olds, *p* < 0.001; Table 3). This was particularly true for HFSS products (65% in 18–34 year olds vs. 31% in 65 + year olds, *p* < 0.01; OSM Table S3). Female respondents observed slightly less advertising than males (56% vs. 61%, *p* = 0.09; Table 3) and white respondents observed slightly less advertising than non-white respondents (58% vs. 66%, p0.03; Table 3); this pattern was more evident in Bristol (65% vs 77%) than South Gloucestershire (52% vs. 53%; Table 3). Respondents living in more deprived areas observed more advertising than respondents in less deprived areas (64% in deciles 1–2 vs. 52% in deciles 9–10, *p* < 0.01; Table 3); this was particularly true for HFSS advertising (50% in deciles 1–2 vs. 29% in deciles 9–10, *p* < 0.01; OSM Table S3). Respondents who used the bus more often were more likely to see advertising than those who used the bus less often (*p* < 0.01, Table 3).

**Table 3:** Self-reported advert exposure by respondent characteristics

	**Bristol (*n* = 1,110)**		**South Gloucestershire (*n* = 1,433)**		**Overall (*n* = 2,543)**		
	**n**	**%**	**n**	**n**	**%**	**n**	***p*-value**
**Age**							*p* < 0.01
18–34 years	140/170	82.4%	52/78	66.7%	192/248	77.4%	
35–44 years	127/159	79.9%	61/104	58.7%	188/263	71.5%	
45–64 years	251/427	58.8%	255/476	53.6%	506/903	56.0%	
65 + years	208/342	60.8%	371/760	48.8%	579/1102	52.5%	
**Sex**							*p* = 0.09
Female	403/620	65.0%	439/876	50.1%	842/1496	56.3%	
Male	307/457	67.2%	299/542	55.2%	606/999	60.7%	
Other	4/5	80.0%	0/1	0.0%	4/6	66.7%	
**Ethnicity**							*p* = 0.03
White	639/982	65.1%	669/1292	51.8%	1308/2274	57.5%	
Non-white	73/95	76.8%	46/86	53.5%	119/181	65.7%	
**IMD decile**							*p* < 0.01
1–2 (most deprived)	422/656	64.3%	4/8	50.0%	426/664	64.2%	
3–4	108/138	78.3%	108/185	58.4%	216/323	66.9%	
5–6	72/107	67.3%	111/226	49.1%	183/333	55.0%	
7–8	87/125	69.6%	199/387	51.4%	286/512	55.9%	
9–10 (least deprived)	44/84	52.4%	326/627	52.0%	370/711	52.0%	
**Bus use**							*p* < 0.01
Daily	56/76	73.7%	0/2	0.0%	56/78	71.8%	
Several times per week	105/143	73.4%	37/61	60.7%	142/204	69.6%	
Several times per month	152/213	71.4%	99/156	63.5%	251/369	68.0%	
Once per month or less	242/384	63.0%	341/631	54.0%	583/1015	57.4%	
Never	170/285	59.6%	260/558	46.6%	430/843	51.0%	

*IMD* Index of Multiple Deprivation

*p* values derived from Chi-square tests

## Discussion

This mixed-methods study aimed to describe the perceived advertising environment prior to the implementation of the new advertisement policy and explore barriers and facilitators for the implementation and planning of the policy.

Prior to the implementation of the new policy (i.e. during its development), 58% of respondents residing in Bristol and South Gloucestershire reported noticing advertisements for unhealthy commodities in the week prior to completing the survey. This was highest for HFSS products (40%), while 16% of residents observed HFSS product advertisements aimed at children. This was lower than reported in London (85%) [9], but slightly higher than observed by researchers for a city in the North of England, where 49% of advertisements depicted food and beverages, with 35% of these considered less healthy [8]. For HFSS products, in particular, younger people were more likely to observe adverts than older people, as were those who were from more deprived areas (compared to those from less deprived areas). With respect to the target area of the new policy, Bristol residents were more likely to see advertising than South Gloucestershire residents. The finding that self-reported exposure to unhealthy commodity advertisements was higher in more deprived areas, and in younger people reported, was also observed in London before the implementation of the HFSS advertisement policy [9], as well as elsewhere nationally and internationally [7, 23–25]; but not everywhere [8, 24]. Findings from Bristol corroborate previous evidence from other areas indicating that young people and people living in lower socio-economic areas are exposed to, or observe, more advertising of HFSS products, but that there is less evidence of such a correlation for other unhealthy commodities; notably alcohol, gambling and payday loan advertisements. A correlation with ethnicity in which ethnic minorities were exposed to more unhealthy commodities advertising than people of white ethnicity was observed elsewhere [26], was identified in Bristol but not South Gloucestershire.

These data indicate that an advertisement policy that restricts the advertisement of such unhealthy commodities, and in particular for HFSS products, has the potential to differentially impact on less-advantaged population subgroups, and thus have the potential to reduce health inequalities. Evidence from the TfL HFSS policy suggests that this might indeed be the case [27]. This rationale directly influenced the development of the advertisement policy in Bristol. Indeed, interviews with policmakers indicated how the policy benefitted from an existing supportive environment following the ‘health in all policies’ initiative and a focus on reducing health inequalities across the city, which was supported by the city Mayor. This was further supported by the precedent set by the TfL HFSS advertisement policy. However, despite this focus and support, initial barriers to implementation of the policy included concerns about how the policy might negatively impact other areas such as the night-time economy and other commercial activities that benefit the city. There were also some concerns that the policy might not lead to measurable impacts as the council only owned about 30% of the advertisement estate. These insights resonate with those of the TfL policy [28], and are particularly useful for other councils who may also be looking to implement a similar policy. At the time of writing, about 80 councils in England have expressed an interest, and in addition to Bristol, the town of Barnsley has also implemented such a policy [29]. Wales is similarly aiming to implement a comparable policy as part of its ‘Healthy Weight: Healthy Wales’ plan [30].

This study had several strengths. The survey sample size was relatively large with more than 2,500 respondents’ data included in the analysis. Efforts were made to sample from a wide range of individuals, including those who are sometimes harder to reach. By providing paper copies as well as online questionnaires, we were able to better reach people without internet access or who find technology hard to work with. Because the research was developed in close collaboration with the councils, we were able to capitalise on existing mechanisms for dissemination of the survey. As a result, we were able to conduct semi-structured interviews with the key stakeholders involved in designing and implementing the policy without the need to resort to snowballing. However, the study also has several limitations. We had a relatively small sample size for the interviews. We believe that those that were interviewed included the important stakeholders involved in the development and implementation of the policy; nonetheless, future work would benefit from the inclusion of a larger group of interviewees, including those involved in the periphery of the policy development process. Respondents from Bristol and South Gloucestershire differed in terms of age and social deprivation distributions, with those in Bristol being younger and living in more deprived areas, on average. This mostly reflects the true differences between residents of the two areas but does not facilitate straightforward comparisons between them. Further, the sample population is older, and contains a larger percentage of women compared to the general populations in the two areas. Because of the sampling strategy, it is further not possible to determine the survey response rates in both areas, as the denominator is unknown. The evaluation of advertisement exposure was based on self-reported rather than objectively measured exposure, which will have introduced measurement error. This may be compounded by the lack of precision in the definition of ‘local area’ that we used and the likely issues with recall for a specific period (i.e. in the last week). Nonetheless, we observe a moderate correlation between reported and measured advertisement exposure implying this is a useful measure to use. Arguably, it is also important to measure what advertising people recall seeing in what they consider their neighbourhood or local area rather than measuring potential advertisement exposure that residents might not have noticed. Future studies should explore this in more detail.

In conclusion, this study supports evidence from other regions of the UK that unhealthy commodities advertisements are observed more by younger people and people in more deprived areas, especially where the advertisement of products high in fat, sugar or salts and fast-food brands are concerned. Policies that specifically restrict such advertisements, therefore, have the potential to reduce health inequalities. The specific Bristol context supported the successful development and implementation of such a policy. Future evaluation of the policy will provide evidence of whether such policies can have a measurable impact on public health and reduce health inequalities, in particular in a location outside of London where the council only owns part of the advertisement estate.

## Supplementary Information

**Additional file 1: Table S1.** Stakeholder topic guide. **Table S2.** Survey questions HFSS, alcohol and gambling modules. **Table S3.** Self-reported exposure to different types of adverts by respondent characteristics.

## Data Availability

The datasets generated and/or analysed during the current study are subject to a data sharing mandate but are available from the corresponding author on reasonable request.
