# Supplementary material for: Advertisement of unhealthy commodities in Bristol and South Gloucestershire and rationale for a new advertisement policy
Source: BMC Public Health. 2023 Jun 5;23:1078. doi: 10.1186/s12889-023-15995-z (PMC10242802; doi:10.1186/s12889-023-15995-z)
Supplement: Supplementary file 1 — Additional file 1: Table S1. Stakeholder topic guide. Table S2. Survey questions HFSS, alcohol and gambling modules. Table S3. Self-reported exposure to different types of adverts by respondent characteristics. [file 12889_2023_15995_MOESM1_ESM.pdf]

# Advertisement of unhealthy commodities in Bristol and South Gloucestershire and rationale for a new advertisement policy

## ONLINE SUPPLEMENTARY MATERIALS

**Table S1: Stakeholder topic guide**

| | |
|---|---|
| **1.** | **Involvement** \| How have you been involved in the development of the Bristol Advertising and Sponsorship Policy over the last few years? |
| **2.** | **History of the policy** \| What lead to the banning of unhealthy commodity advertising being included in this version of the policy?<br>• *Prompt*: Why was the decision made to restrict advertising of high fat, salt and sugar (HFSS) food and drink products, alcohol, and gambling in public places and council owned premises?<br>• *Probe*: Was there anything else that led to, or influenced, these decisions being made to focus on banning the advertisement of unhealthy commodities? |
| **3.** | **Planning the policy** \| Could you talk me through the process of planning the advertising ban?<br>• *Prompt*: What steps were followed to plan the policy?<br>• *Probe*: Could you describe what resources were required to plan this policy? (e.g. people, time, consultancy fees, consultation exercises, etc?)<br>• *Probe*: Were any frameworks or evidence used to support the planning?<br>• *Probe*: Was there any public consultation around this and what did that look like? |
| **4.** | **Anticipated impacts on the system** \| From your perspective, what are the perceived benefits or expected outcomes of the advertising ban?<br>• *Prompt*: In other words, what changes do you expect to see locally that may be associated with the advertising ban?<br>• *Probe*: How are you planning to monitor the impact / evaluate the policy?<br>• *Probe:* Do you have any specific targets relating to outcome measures?<br>• *Probe*: When do you anticipate seeing some of these benefits?<br>• *Probe*: Do you expect any loss of advertisement revenue, a reduction in tax revenue or any other economic consequences after implementing this policy?<br>• *Probe*: Are there any wider or unintended impacts that you anticipate might occur due to the implementation of the advertising ban? |
| **5.** | **Actual barriers and facilitators** \| What learnings, if any, have come from the process of planning the advertising ban?<br>• *Prompt*: What has helped with the planning of the ban or made it work well?<br>• *Prompt*: What challenges or barriers were experienced when planning the advertising ban?<br>• *Probe*: Were you able to overcome these challenges? If so, how did you work around them? |
| **6.** | **Future plans for the policy \|** What are the future plans for the policy?<br>• *Probe*: What do you anticipate will help and hinder the future success of the advertising ban?<br>• *Probe*: Is there anything that needs to be put in place to support or mitigate this occurring? |
| **7.** | **Anything else** \| Is there anything else that you would like to mention regarding the forthcoming advertising ban? |
| **8.** | **Snowballing** \| Do you have any recommendations on who else might be useful to speak with about the planning of this policy? |

## Table S2: Survey questions HFSS, alcohol and gambling modules.

**Foods and drinks High in Fat, Sugar and/or Salt (HFSS)**

1. In the last week, did you see advertisements for these types of foods and drinks in your local area? (tick all that apply)

 □ I don't remember seeing adverts for products in the list below
 □ chocolate and confectionary
 □ biscuits and cakes
 □ desserts (like ice cream, puddings)
 □ sugary cereals
 □ crisps & savoury snacks (like crisps, crackers, popcorn, poppadums, prawn crackers…)
 □ fast food (burgers, burritos…)
 □ sugary drinks (cola, sprite,…)

10. If you answered yes to the question above, do you remember where you saw the advertisements for these foods (tick all that apply)?
□ No
□ Yes, on
 □ a billboard
 □ a bus stop
 □ the side of a bus
 □ elsewhere, please add where: ……………………………………..
 □ no, I do not remember where

In the last week, which of the following food chains have you seen advertised?
(Tick all that apply)
 □ I don't remember seeing adverts for the chains in the list below
 □ McDonald's
 □ Burger King
 □ KFC
 □ Subway
 □ Dominos
 □ Pappa Johns
 □ Nando's
 □ Greggs
 □ Pret a Manger
 □ Coffee chains (such as Costa, Nero, Starbucks, etc)
 □ Other, please write ………………………………………

11. If you answered yes to the question above, do you remember where you saw the advertisement?
□ No
□ Yes, on
 □ a billboard
 □ a bus stop
 □ the side of a bus
 □ elsewhere, please add where: ……………………………………..
 □ no, I do not remember where

12. In the last week, have you seen any ads for HFSS **OR** fast food, confectionery, or soft drinks you believe to be specifically aimed at children in your community?
□ No
□ Yes, specifically
 □ chocolate and confectionary
 □ pudding and biscuits
 □ sugary cereals
 □ sugary snacks (crisps, crackers, popcorn, poppadums, prawn crackers…)
 □ fastfood (burgers, burritos…)
 □ softdrinks

13. If you answered yes to the above question, do you remember where you saw the advertisement(s) specifically aimed at children?

□ No
□ Yes, on
- □ a billboard
- □ a bus stop
- □ the side of a bus
- □ elsewhere, please add where: ………………………………………..
- □ no, I do not remember where

14. In the last week, have you used any of the products mentioned in the questions above?
(tick all that apply)
- □ No, I have not used any of these
- □ I don't remember

Yes,
- □ chocolate or confectionary
- □ biscuits or cakes
- □ desserts (like ice cream, puddings)
- □ sugary cereals
- □ crisps & savoury snacks (like crisps, crackers, popcorn, poppadums, prawn crackers…)
- □ fast food (burgers, burritos…)
- □ sugary drinks

**ALCOHOL**

15. In the last week, do you remember seeing any advertisements for alcoholic drinks in your community?
(tick all that apply)

□ No
□ Yes, specifically
- □ beer
- □ wine
- □ liquor (whiskey, gin, vodka…)
- □ mixtures (breezers…)

16. If you answered yes to the above, do you remember where you saw the advertisement for alcoholic drinks?
□ No
□ Yes, on
- □ a billboard
- □ a bus stop
- □ the side of a bus
- □ elsewhere, please add where: ………………………………………..
- □ no, I do not remember where

17. In the last week, do you remember seeing any advertisements of places where alcohol can be consumed, such as pubs and restaurants, in your community?
(tick all that apply)

□ No
□ Yes, specifically
- □ a pub, or a chain (for example Weatherspoons)
- □ a restaurant
- □ other, namely:……………………………………

18. In the last week, have you drank any alcoholic drinks?
(tick all that apply)
- □ No
- □ I cannot remember

Yes,
- □ beer
- □ wine

□ liquor (whiskey, gin, vodka…)
□ mixtures (breezers…)

19. In the last week, have you been to a pub or restaurant and consumed any alcoholic drink?
(tick all that apply)
□ No
□ I cannot remember

Yes,
□ pub
□ restaurant
□ other, namely……………………………..

**Gambling**

20. In the last week, do you remember seeing advertisements by any of the following gambling companies or websites in your community?
(Tick all that apply)

□ I don't remember seeing adverts for the companies or websites in the list below
□ Sport Betting
□ Ladbrokes
□ Betfair
□ Paddy Power
□ William Hill
□ National Lottery
□ a Casino
□ a Bingo venue
□ online gambling sites (including Bingo, 777, Poker or Casino sites)
□ a racecourse (Chester and Cheltenham racecourses, for example)
□ Other, please write …………………………………………

21. If you answered yes to the above, do you remember where you saw the advertisements for gambling companies or websites?
□ No
□ Yes, on
□ a billboard
□ a bus stop
□ the side of a bus
□ elsewhere, please add where: ………………………………………..
□ no, I do not remember where

22. In the last week, have you done any gambling?
(tick all that apply)
□ No
□ I cannot remember

Yes,
□ in a bookmaker/bookies
□ online (for example Bingo, poker or casino games, sportsbetting)
□ in a shop (including lottery, scratch cards)
□ in a casino
□ in a Bingo venue
□ in a racecourse (Chester and Cheltenham racecourses, for example)
□ somewhere else, please write …………………………………………

**Table S3 Self-reported exposure to different types of adverts by respondent characteristics**

| | Bristol (n=1,110) | | South Gloucestershire (n=1,433) | | Overall (n=2,543) | | |
|---|---|---|---|---|---|---|---|
| | n | % | n | n | % | n | p-value |
| **Age** | | | | | | | |
| **Any ads** | | | | | | | P<0.001 |
| 18-34 years | 140/170 | 82.4% | 52/78 | 66.7% | 192/248 | 77.4% | |
| 35-44 years | 127/159 | 79.9% | 61/104 | 58.7% | 188/263 | 71.5% | |
| 45-64 years | 251/427 | 58.8% | 255/476 | 53.6% | 506/903 | 56.0% | |
| 65+ years | 208/342 | 60.8% | 371/760 | 48.8% | 579/1102 | 52.5% | |
| **HFSS** | | | | | | | P<0.001 |
| 18-34 years | 128/170 | 75.3% | 34/78 | 43.6% | 162/248 | 65.3% | |
| 35-44 years | 111/159 | 69.8% | 42/104 | 40.4% | 153/263 | 58.2% | |
| 45-64 years | 198/427 | 46.4% | 160/476 | 33.6% | 358/903 | 39.6% | |
| 65+ years | 144/342 | 42.1% | 203/760 | 26.7% | 347/1102 | 31.5% | |
| **HFSS for children** | | | | | | | P<0.001 |
| 18-34 years | 35/170 | 20.6% | 10/78 | 12.8% | 45/248 | 18.1% | |
| 35-44 years | 58/159 | 36.5% | 15/104 | 14.4% | 73/263 | 27.8% | |
| 45-64 years | 88/427 | 20.6% | 54/476 | 11.3% | 142/903 | 15.7% | |
| 65+ years | 71/342 | 20.8% | 75/760 | 9.9% | 146/1102 | 13.2% | |
| **Alcohol** | | | | | | | P=0.034 |
| 18-34 years | 42/170 | 24.7% | 10/78 | 12.8% | 52/248 | 21.0% | |
| 35-44 years | 36/159 | 22.6% | 19/104 | 18.3% | 55/263 | 20.9% | |
| 45-64 years | 81/427 | 19.0% | 58/476 | 12.2% | 139/903 | 15.4% | |
| 65+ years | 73/342 | 21.3% | 99/760 | 13.0% | 172/1102 | 15.6% | |
| **Establishments selling alcohol** | | | | | | | P=0.895 |
| 18-34 years | 34/170 | 20.0% | 20/78 | 25.6% | 54/248 | 21.8% | |
| 35-44 years | 27/159 | 17.0% | 30/104 | 28.8% | 57/263 | 21.7% | |
| 45-64 years | 70/427 | 16.4% | 113/476 | 23.7% | 183/903 | 20.3% | |
| 65+ years | 63/342 | 18.4% | 174/760 | 22.9% | 237/1102 | 21.5% | |
| **Gambling** | | | | | | | P=0.286 |
| 18-34 years | 60/170 | 35.3% | 16/78 | 20.5% | 76/248 | 30.6% | |
| 35-44 years | 61/159 | 38.4% | 24/104 | 23.1% | 85/263 | 32.3% | |
| 45-64 years | 128/427 | 30.0% | 114/476 | 23.9% | 242/903 | 26.8% | |
| 65+ years | 119/342 | 34.8% | 191/760 | 25.1% | 310/1102 | 28.1% | |
| **Sex** | | | | | | | |
| **Any ads** | | | | | | | P=0.086 |
| Female | 403/620 | 65.0% | 439/876 | 50.1% | 842/1496 | 56.3% | |
| Male | 307/457 | 67.2% | 299/542 | 55.2% | 606/999 | 60.7% | |
| Other | 4/5 | 80.0% | 0/1 | 0.0% | 4/6 | 66.7% | |
| **HFSS** | | | | | | | P=0.468 |
| Female | 329/620 | 53.1% | 260/876 | 29.7% | 589/1496 | 39.4% | |
| Male | 239/457 | 52.3% | 177/542 | 32.7% | 416/999 | 41.6% | |
| Other | 3/5 | 60.0% | 0/1 | 0.0% | 3/6 | 50.0% | |
| **HFSS for children** | | | | | | | P=0.003 |
| Female | 128/620 | 20.6% | 82/876 | 9.4% | 210/1496 | 14.0% | |
| Male | 118/457 | 25.8% | 71/542 | 13.1% | 189/999 | 18.9% | |
| Other | 0/5 | 0.0% | 0/1 | 0.0% | 0/6 | 0.0% | |
| **Alcohol** | | | | | | | P<0.001 |

| | | | | | | | |
|---|---|---|---|---|---|---|---|
| Female | 116/620 | 18.7% | 91/876 | 10.4% | 207/1496 | 13.8% | |
| Male | 109/457 | 23.9% | 92/542 | 17.0% | 201/999 | 20.1% | |
| Other | 4/5 | 80.0% | 0/1 | 0.0% | 4/6 | 66.7% | |
| **Establishments selling alcohol** | | | | | | | P=0.133 |
| Female | 101/620 | 16.3% | 194/876 | 22.1% | 295/1496 | 19.7% | |
| Male | 86/457 | 18.8% | 142/542 | 26.2% | 228/999 | 22.8% | |
| Other | 2/5 | 40.0% | 0/1 | 0.0% | 2/6 | 33.3% | |
| **Gambling** | | | | | | | P<0.001 |
| Female | 191/620 | 30.8% | 185/876 | 21.1% | 376/1496 | 25.1% | |
| Male | 169/457 | 37.0% | 161/542 | 29.7% | 330/999 | 33.0% | |
| Other | 1/5 | 20.0% | 0/1 | 0.0% | 1/6 | 16.7% | |
| **Ethnicity** | | | | | | | |
| **Any ads** | | | | | | | P=0.031 |
| White | 639/982 | 65.1% | 669/1292 | 51.8% | 1308/2274 | 57.5% | |
| Non-white | 73/95 | 76.8% | 46/86 | 53.5% | 119/181 | 65.7% | |
| **HFSS** | | | | | | | P=0.057 |
| White | 506/982 | 51.5% | 398/1292 | 30.8% | 904/2274 | 39.8% | |
| Non-white | 59/95 | 62.1% | 26/86 | 30.2% | 85/181 | 47.0% | |
| **HFSS for children** | | | | | | | P=0.030 |
| White | 213/982 | 21.7% | 138/1292 | 10.7% | 351/2274 | 15.4% | |
| Non-white | 28/95 | 29.5% | 11/86 | 12.8% | 39/181 | 21.5% | |
| **Alcohol** | | | | | | | P=0.142 |
| White | 202/982 | 20.6% | 167/1292 | 12.9% | 369/2274 | 16.2% | |
| Non-white | 25/95 | 26.3% | 12/86 | 14.0% | 37/181 | 20.4% | |
| **Establishments selling alcohol** | | | | | | | P=0.575 |
| White | 171/982 | 17.4% | 304/1292 | 23.5% | 475/2274 | 20.9% | |
| Non-white | 17/95 | 17.9% | 24/86 | 27.9% | 41/181 | 22.7% | |
| **Gambling** | | | | | | | P=0.038 |
| White | 318/982 | 32.4% | 310/1292 | 24.0% | 628/2274 | 27.6% | |
| Non-white | 39/95 | 41.1% | 24/86 | 27.9% | 63/181 | 34.8% | |
| **IMD decile** | | | | | | | |
| **Any ads** | | | | | | | P<0.001 |
| 1-2 (most deprived) | 422/656 | 64.3% | 4/8 | 50.0% | 426/664 | 64.2% | |
| 3-4 | 108/138 | 78.3% | 108/185 | 58.4% | 216/323 | 66.9% | |
| 5-6 | 72/107 | 67.3% | 111/226 | 49.1% | 183/333 | 55.0% | |
| 7-8 | 87/125 | 69.6% | 199/387 | 51.4% | 286/512 | 55.9% | |
| 9-10 (least deprived) | 44/84 | 52.4% | 326/627 | 52.0% | 370/711 | 52.0% | |
| **HFSS** | | | | | | | P<0.001 |
| 1-2 (most deprived) | 330/656 | 50.3% | 3/8 | 37.5% | 333/664 | 50.2% | |
| 3-4 | 91/138 | 65.9% | 63/185 | 34.1% | 154/323 | 47.7% | |
| 5-6 | 61/107 | 57.0% | 67/226 | 29.6% | 128/333 | 38.4% | |
| 7-8 | 72/125 | 57.6% | 132/387 | 34.1% | 204/512 | 39.8% | |
| 9-10 (least deprived) | 30/84 | 35.7% | 179/627 | 28.5% | 209/711 | 29.4% | |
| **HFSS for children** | | | | | | | P<0.001 |

| | | | | | | | |
|---|---|---|---|---|---|---|---|
| 1-2 (most deprived) | 155/656 | 23.6% | 1/8 | 12.5% | 156/664 | 23.5% | |
| 3-4 | 35/138 | 25.4% | 25/185 | 13.5% | 60/323 | 18.6% | |
| 5-6 | 25/107 | 23.4% | 22/226 | 9.7% | 47/333 | 14.1% | |
| 7-8 | 23/125 | 18.4% | 44/387 | 11.4% | 67/512 | 13.1% | |
| 9-10 (least deprived) | 16/84 | 19.0% | 64/627 | 10.2% | 80/711 | 11.3% | |
| **Alcohol** | | | | | | | P<0.001 |
| 1-2 (most deprived) | 145/656 | 22.1% | 1/8 | 12.5% | 146/664 | 22.0% | |
| 3-4 | 28/138 | 20.3% | 24/185 | 13.0% | 52/323 | 16.1% | |
| 5-6 | 16/107 | 15.0% | 28/226 | 12.4% | 44/333 | 13.2% | |
| 7-8 | 32/125 | 25.6% | 51/387 | 13.2% | 83/512 | 16.2% | |
| 9-10 (least deprived) | 13/84 | 15.5% | 82/627 | 13.1% | 95/711 | 13.4% | |
| **Establishments selling alcohol** | | | | | | | P=0.003 |
| 1-2 (most deprived) | 106/656 | 16.2% | 1/8 | 12.5% | 107/664 | 16.1% | |
| 3-4 | 27/138 | 19.6% | 52/185 | 28.1% | 79/323 | 24.5% | |
| 5-6 | 18/107 | 16.8% | 48/226 | 21.2% | 66/333 | 19.8% | |
| 7-8 | 26/125 | 20.8% | 92/387 | 23.8% | 118/512 | 23.0% | |
| 9-10 (least deprived) | 19/84 | 22.6% | 147/627 | 23.4% | 166/711 | 23.3% | |
| **Gambling** | | | | | | | P<0.001 |
| 1-2 (most deprived) | 229/656 | 34.9% | 0/8 | 0.0% | 229/664 | 34.5% | |
| 3-4 | 58/138 | 42.0% | 55/185 | 29.7% | 113/323 | 35.0% | |
| 5-6 | 29/107 | 27.1% | 50/226 | 22.1% | 79/333 | 23.7% | |
| 7-8 | 37/125 | 29.6% | 96/387 | 24.8% | 133/512 | 26.0% | |
| 9-10 (least deprived) | 19/84 | 22.6% | 151/627 | 24.1% | 170/711 | 23.9% | |
| **Bus use** | | | | | | | |
| **Any ads** | | | | | | | P<0.001 |
| Daily | 56/76 | 73.7% | 0/2 | 0.0% | 56/78 | 71.8% | |
| Several times per week | 105/143 | 73.4% | 37/61 | 60.7% | 142/204 | 69.6% | |
| Several times per month | 152/213 | 71.4% | 99/156 | 63.5% | 251/369 | 68.0% | |
| Once per month or less | 242/384 | 63.0% | 341/631 | 54.0% | 583/1015 | 57.4% | |
| Never | 170/285 | 59.6% | 260/558 | 46.6% | 430/843 | 51.0% | |
| **HFSS** | | | | | | | P<0.001 |
| Daily | 44/76 | 57.9% | 0/2 | 0.0% | 44/78 | 56.4% | |
| Several times per week | 85/143 | 59.4% | 24/61 | 39.3% | 109/204 | 53.4% | |
| Several times per month | 127/213 | 59.6% | 64/156 | 41.0% | 191/369 | 51.8% | |
| Once per month or less | 194/384 | 50.5% | 199/631 | 31.5% | 393/1015 | 38.7% | |
| Never | 129/285 | 45.3% | 147/558 | 26.3% | 276/843 | 32.7% | |

| | | | | | | | |
|---|---|---|---|---|---|---|---|
| **HFSS for children** | | | | | | | P<0.001 |
| Daily | 30/76 | 39.5% | 0/2 | 0.0% | 30/78 | 38.5% | |
| Several times per week | 45/143 | 31.5% | 14/61 | 23.0% | 59/204 | 28.9% | |
| Several times per month | 50/213 | 23.5% | 24/156 | 15.4% | 74/369 | 20.1% | |
| Once per month or less | 63/384 | 16.4% | 56/631 | 8.9% | 119/1015 | 11.7% | |
| Never | 63/285 | 22.1% | 53/558 | 9.5% | 116/843 | 13.8% | |
| **Alcohol** | | | | | | | P<0.001 |
| Daily | 21/76 | 27.6% | 0/2 | 0.0% | 21/78 | 26.9% | |
| Several times per week | 41/143 | 28.7% | 15/61 | 24.6% | 56/204 | 27.5% | |
| Several times per month | 58/213 | 27.2% | 25/156 | 16.0% | 83/369 | 22.5% | |
| Once per month or less | 73/384 | 19.0% | 86/631 | 13.6% | 159/1015 | 15.7% | |
| Never | 39/285 | 13.7% | 57/558 | 10.2% | 96/843 | 11.4% | |
| **Establishments selling alcohol** | | | | | | | P=0.590 |
| Daily | 18/76 | 23.7% | 0/2 | 0.0% | 18/78 | 23.1% | |
| Several times per week | 32/143 | 22.4% | 13/61 | 21.3% | 45/204 | 22.1% | |
| Several times per month | 41/213 | 19.2% | 38/156 | 24.4% | 79/369 | 21.4% | |
| Once per month or less | 65/384 | 16.9% | 160/631 | 25.4% | 225/1015 | 22.2% | |
| Never | 37/285 | 13.0% | 125/558 | 22.4% | 162/843 | 19.2% | |
| **Gambling** | | | | | | | P<0.001 |
| Daily | 27/76 | 35.5% | 0/2 | 0.0% | 27/78 | 34.6% | |
| Several times per week | 64/143 | 44.8% | 23/61 | 37.7% | 87/204 | 42.6% | |
| Several times per month | 75/213 | 35.2% | 54/156 | 34.6% | 129/369 | 35.0% | |
| Once per month or less | 115/384 | 29.9% | 160/631 | 25.4% | 275/1015 | 27.1% | |
| Never | 89/285 | 31.2% | 108/558 | 19.4% | 197/843 | 23.4% | |
